# Sub‑2 nm Equivalent-Oxide-Thickness Ferroelectric Transistors for Cryogenic Memory and Computing

**DOI:** 10.1021/acsnano.5c16255

**Published:** 2026-03-31

**Authors:** Apu Das, Asim Senapati, Gautham Kumar, Zhao-Feng Lou, Jonas Müller, Jaskirat Singh Maskeen, Yii-Tay Chang, Mohit Tewari, Ankit Agarwal, Agniva Paul, Yannick Raffel, Siddheswar Maikap, Kuo-Hsing Kao, Tarun Agarwal, Sandip Lashkare, Darsen Lu, Guilhem Larrieu, Min-Hung Lee, Sourav De

**Affiliations:** † College of Semiconductor Research (CoSR), National Tsing Hua University (NTHU), Hsinchu 300044, Taiwan; ‡ Graduate Institute of Electronics Engineering (GIEE), National Taiwan University (NTU), Taipei 106319, Taiwan; § Laboratory for Analysis and Architecture of Systems (LAAS-CNRS), Université de Toulouse, 31031 Toulouse, France; ∥ Department of Computer Science and Engineering, 242275 Indian Institute of Technology Gandhinagar, Palaj, Gujarat 382055, India; ⊥ Department of Electrical Engineering, Indian Institute of Technology Gandhinagar, Palaj, Gujarat 382055, India; # Department of Electrical Engineering, National Cheng Kung University (NCKU), Tainan 70101, Taiwan; ∇ Fraunhofer Institute for Photonic Microsystems IPMS—Center Nanoelectronic Technologies, 01109 Dresden, Germany; ○ Department of Electronic Engineering, Chang Gung University (CGU), Taoyuan 333323, Taiwan

**Keywords:** FeFETs, HZO, cryogenic electronics, nonvolatile memory, neuromorphic computing, 4D-STEM, XPS

## Abstract

Ferroelectric hafnia-based field-effect transistors are promising candidates for nonvolatile memory and in-memory computing. However, their operation principle under deep-cryogenic conditions at aggressively scaled gate stacks remains underexplored, especially for bulk silicon technology. This work presents an experimental demonstration of front-end-of-line bulk silicon-channel ferroelectric field-effect transistors featuring sub-2 nm equivalent-oxide-thickness gate stacks with ≃5 nm hafnium–zirconium oxide, exhibiting robust switching at 10 K. Key metrics include memory windows exceeding 1 V, tightly distributed threshold voltages (standard deviation ≲ 40 mV), endurance surpassing 10^7^ cycles, and retention projections consistent with decade-scale stability. Correlative four-dimensional scanning transmission electron microscopy phase mapping reveals an increased orthorhombic ferroelectric fraction following electrical wake-up at cryogenic temperatures, correlated with enhanced polarization stability and strengthened oxygen–metal coordination. We hypothesize that suppressed trapping-related instability, along with a higher orthorhombic phase, jointly contribute to this effect. Current–voltage sweeps define an operational design window, with memory-window saturation beyond ±5 V programming voltages and ≳900 ns pulse widths, consistent with nucleation-limited reversal kinetics in ultrathin films. A spiking neural network implemented at 10 K achieves >92% classification accuracy on MNIST and 73.8% accuracy on NMNIST data sets, demonstrating practical utility. These findings provide materials- and device-level insights into scaled hafnia FeFETs for energy-efficient cryogenic applications, including potential integration in quantum–classical systems.

## Graphical abstract

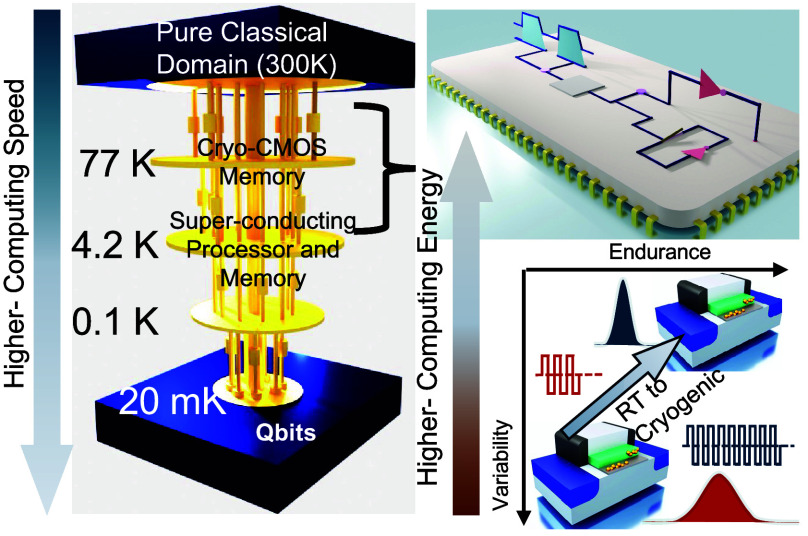

Nonvolatile ferroelectric field-effect transistors (FeFETs) based on hafnia have re-emerged as promising contenders for embedded nonvolatile memory and in-memory computing. Their appeal lies in the unique combination of CMOS compatibility, voltage-driven programming, and low-energy readout, all realized in a simple one-transistor cell.^1–17^ Despite rapid progress at room temperature (RT), two frontiers remain insufficiently resolved for system integration at advanced nodes and in extreme environments. The first is gate-stack scaling toward sub-2 nm effective-oxide thickness (EOT), where depolarization, interfacial dipoles, and leakage through the interfacial oxide (IL) can undermine retention and variability. The second is deep-cryogenic operation (*T* ≲ 10–77 K), increasingly relevant for cryo-CMOS controllers of quantum processors, superconducting logic, and low-noise sensing pipelines. Achieving robust, reproducible ferroelectric switching under both constraints is essential if FeFETs are to provide dense cryogenic memory and synaptic primitives in the quantum–classical stack.^8,10,18–29^

FeFET is particularly attractive at advanced nodes because its noncentrosymmetric orthorhombic phase (Pca2_1_) persists in nanometric films and can be stabilized through stress, grain-size engineering, oxygen-vacancy control, and electrode work-function tuning.^4,7,27,30–32,32–37^ Yet pushing the gate stack to sub-2 nm EOT magnifies the influence of the IL and depolarization field, increases sensitivity to charge-trapping, and fixed-charge fluctuations.^38–41^ Although prior studies at low temperature report steeper subthreshold slopes and improved carrier mobility in Si-FeFETs, and highly stable endurance and retention in amorphous metal-oxide channel-based FeFETs.^23,42^ A mechanistic connection linking cryogenic switching, wake-up, polarization kinetics in ultrathin HZO, and device reliability remains incomplete and requires further investigation.^6,8,10,19,28,43–49^

Here we address this gap by engineering FeFETs that combine *sub-2 nm EOT* stacks with ∼5 nm HZO and mapping their electrical and physical behavior down to 10 K. Cross-sectional transmission electron microscopy (TEM) confirms the TaN/HZO/SiO_2_/p-Si stack geometry and thickness control, while four-dimensional scanning TEM (4D-STEM) with automated crystallography mapping (ACOM) resolves the monoclinic/orthorhombic distribution. These measurements reveal a cryogenic electrical “wake-up” that increases the orthorhombic fraction, correlating with enhanced polarization stability and narrower device-to-device threshold-voltage (*V*_th_) distributions at 10 K. The 4D-STEM data indicate a higher fraction of the ferroelectric phase following wake-up at cryogenic temperature (10 K). We hypothesize that this enhancement originates from a reduction in internal bias due to suppressed trapping-related instability at low temperature; however, a more detailed investigation is required to validate this mechanism conclusively. The increased ferroelectric phase fraction consequently amplifies the polarization-induced surface-potential shift at the HZO/SiO_2_ interface, thereby improving MW stability without inducing excess leakage through the interfacial layer. Under program/erase (PG–ER) pulses of ±5 V and *t*_pw_ = 500 ns, devices exhibit MW > 1 V at 10 K with tight *V*_th_ statistics. Increasing the programming amplitude *V*_P_ or pulse width *t*_pw_ expands the MW but saturates beyond *V*_P_ ≳ 5 V and *t*_pw_ ≳ 900 ns, consistent with nucleation-limited reversal and domain-wall creep kinetics in the ultrathin ferroelectrics in the cryogenic regime. Band-diagram analysis with a common Fermi level across TaN/HZO/SiO_2_/p-Si clarifies the mechanism: polarization charge (±σ_p_) at the HZO/SiO_2_ interface introduces an additional drop Δϕ ≈ σ_p_ *t*_HZO_/(ε_0_ε_HZO_) that modulates the Si surface potential. Retention projections exceed a decade at 10 K, and endurance surpasses 10^7^ cycles within the same PG–ER scheme. Prior cryogenic FeFET demonstrations, such as the back-end-of-line-compatible oxide-channel device reported in ref 23, have achieved remarkable endurance exceeding 10^10^ cycles with no observable degradation and highly stable retention at 77 and 5 K. This outstanding performance underscores the potential of cryogenic operation to suppress thermally activated defect generation and interface trapping, enabling effective ”unlimited” cycling endurance in ferroelectric memories suitable for last-level cache applications in high-performance computing systems.

Beyond device-level metrics, it is equally important to understand the atomistic origins of the stability of FeFETs with ultrathin EOT at low temperature. Our complementary density functional theory-molecular dynamics (DFT–MD) analysis of the SiO_2_/HZO interface demonstrates that cooling from 300 to 10 K reduces interface trap states and strengthens polarization coupling across the interfacial layer simultaneously. This microscopic suppression of traps provides a clear rationale for the experimentally observed tightening of *V*_th_ distributions and the improved separation of high-threshold-voltage (HVT)/low-threshold-voltage (LVT) states at cryogenic temperature (10 K). By explicitly linking trap energetics, band alignment, and polarization screening to macroscopic MW retention, the atomistic study furnishes a materials-level foundation for the design rules we establish in this work.

Finally, we evaluate system-level relevance through a device-aware neuromorphic demonstration at 10 K. FeFET synaptic weights are encoded by programmable *V*_th_ states and read at low *V*_DS_. A spiking neural network (SNN) trained and inferred on Modified National Institute of Standards and Technology (MNIST) and Neuromorphic Modified National Institute of Standards and Technology (N-MNIST) data sets, parametrized by measured cryogenic switching curves and state distributions, achieves >92% and 73.8% test accuracy, respectively, while adhering to endurance cycling limits.

In summary, we have demonstrated FeFETs with sub-2 nm EOT that operate reliably at 10 K. These devices exhibit robust switching, achieving memory windows exceeding 1 V with *V*_th_ variations below 40 mV across devices and cycles. They sustain endurance of 10^7^ cycles under moderate program–erase stress and show retention characteristics that enable reliable decade-long projections, supported by stable *V*_th_ evolution over 10^4^ s measurements at 10 K. Furthermore, 4D-STEM reveals an increased fraction of the orthorhombic ferroelectric phase, and we successfully demonstrate SNN inference at 10 K with accuracy better than room-temperature benchmarks. Collectively, these findings establish clear materials and operational guidelines—including ultrathin HZO layers, sub-2 nm EOT gate stacks, and optimized program–erase schemes,—for integrating hafnium-based FeFET macros and neuromorphic elements into cryo-CMOS, quantum–classical interfaces, and other extreme-environment applications.

## Results

### Structural Analysis

To identify the microscopic origin of the improved ferroelectric (FE) behavior at low temperature, we combined cross-sectional transmission electron microscopy (TEM), four-dimensional scanning transmission electron microscopy (4D-STEM) with automated crystal-orientation mapping (ACOM), X-ray diffraction (Figure S1) and X-ray photoelectron spectroscopy, XPS, (Figure 2) on the ultrathin (∼5 nm) HZO layers used in our FeFETs. Conventional TEM verifies the structural integrity of the gate stack and the thickness control of the FE film, yielding 5.3 nm across FeFETs (Figure 1a). No voids, delamination, or interfacial roughening are observed at the TaN/HZO and HZO/SiO_2_ boundaries, ruling out obvious morphological causes for the temperature trends discussed below. Phase identification and quantification were performed by 4D-STEM ACOM (Figure 1c,d). In this approach, a nanobeam diffraction pattern is recorded at each scan pixel and indexed against crystallographic libraries to assign a local phase with a confidence metric; pixels with low confidence (diffuse patterns, grain boundaries) are masked to avoid bias. The maps unambiguously resolve the monoclinic (*m*, *P*2_1_/*c*) and polar orthorhombic (*o*, *Pca*2_1_) regions in the ultrathin HZO, consistent with prior observations that solid-solution hafnia–zirconia near 1:1 composition stabilizes multiple polymorphs under the combined influence of interfacial fields and strain. Importantly, correlative measurements before and after electrical wake-up at 10 K show a systematic increase in the orthorhombic fraction: the area fraction assigned to the *o*-phase rises from 21.4% at 300 K to 34.9% after low-temperature wake-up (Figure S2). Because the acquisition and indexing parameters are kept identical across temperatures, this change reflects a genuine redistribution of phase content rather than an analysis artifact.

**Figure 1:**
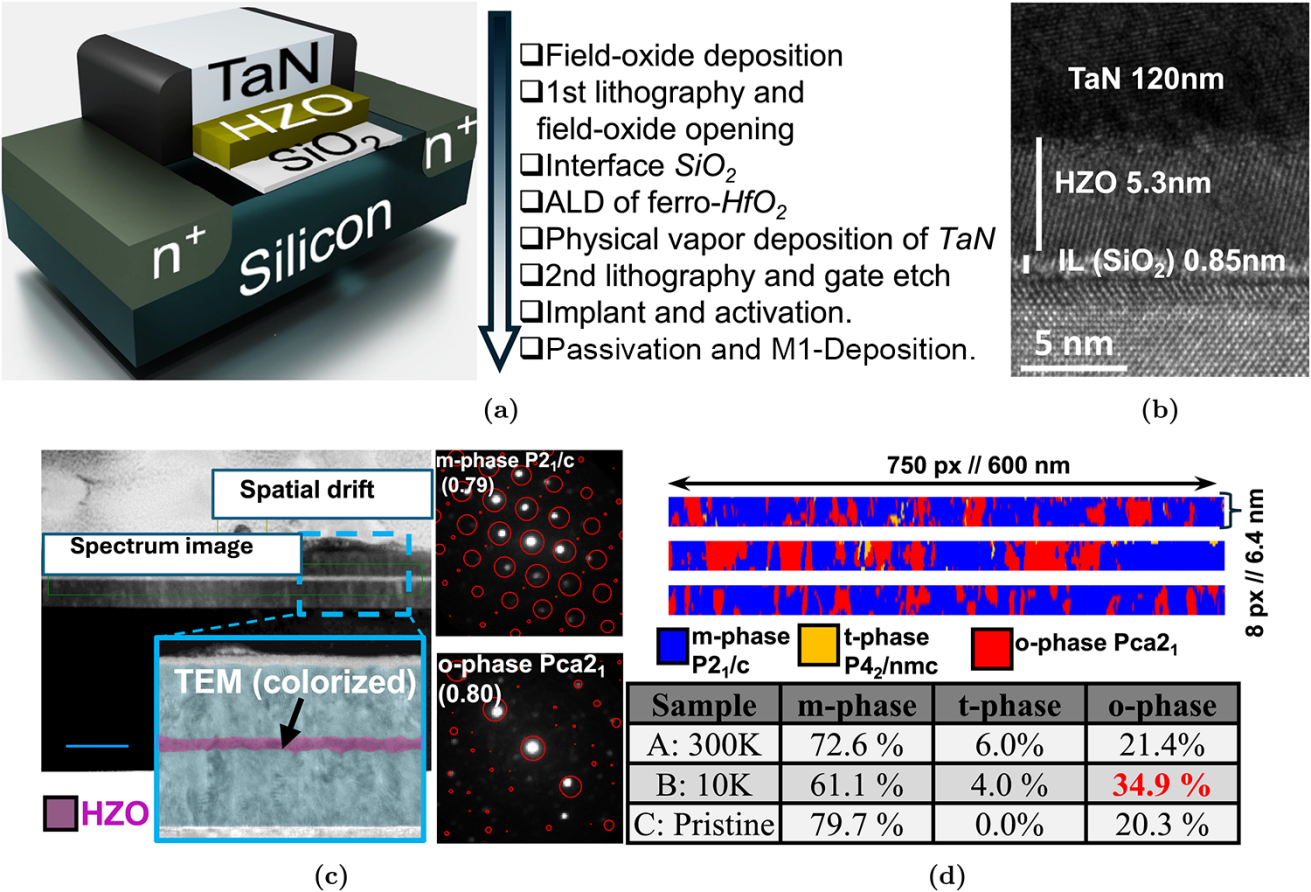
Device stack, process flow, and structural analysis of ultrathin-HZO FeFETs. (a) Fabrication overview and gate-stack (TaN/HZO/SiO_2_/Si). The flow comprises field-oxide patterning, interfacial SiO_2_ formation, atomic-layer deposition (ALD) of Hf_0.5_Zr_0.5_O_2_ (HZO), TaN gate deposition and anisotropic etch, source/drain implantation and activation, and backend metallization. The ultrathin HZO is placed directly above the interfacial SiO_2_ to minimize depolarization and maintain sub-2 nm (1.68 nm) EOT while preserving ferroelectric switching. (b) Cross-sectional TEM confirming uniform layer formation and precise thickness control in representative capacitors/FeFETs: HZO thickness *t*_HZO_ = 5.3 nm and interfacial SiO_2_ thickness *t*_IL_ = 0.85 nm (values from local metrology), evidencing a clean, abrupt TaN/HZO/SiO_2_/Si stack without observable intermixing. (c) 4D-STEM with automated crystal-orientation mapping (ACOM) performed on the 5 nm HZO film. Phase-resolved indexing delineates monoclinic (*m*, *P*2_1_/*c*) and ferroelectric orthorhombic (*o*, *Pca*2_1_) regions at nanometric resolution; high phase-confidence segmentation confirms robust crystallographic discrimination within the scaled film. (d) Temperature- and stimulus-dependent phase-fraction comparison across three states: room temperature (RT, 300 K), cryogenic operation at 10 K following electrical “wake-up” (initial cycling that mobilizes pinned domains/defects), and the pristine state prior to wake-up. Quantitative mapping shows the *o*-phase fraction increasing from 21.4% at RT to 34.9% at 10 K postwake-up, indicating cryo-assisted stabilization of the polar phase.

Although the 4D-STEM analysis probes a localized region of the film, its role in this work is to establish the presence, stability, and spatial continuity of the orthorhombic ferroelectric phase after cryogenic wake-up, rather than to claim enhanced intrinsic ferroelectric switching. Although the 4D-STEM analysis probes a localized region of the film, its role here is to establish the presence and spatial continuity of the orthorhombic ferroelectric phase after cryogenic wake-up, rather than to claim enhanced intrinsic switching strength. Complementary global electrical measurements (PUND, switching-current, and cryogenic *C*–*V*, discussed later) confirm that ferroelectric switching remains active across the full device area at 10 K; however, these data also show that intrinsic polarization and switching strength are not increased at cryogenic temperatures (10 K) within the same voltage range.

We attribute the cryogenic increase in *o*-phase to field-assisted stabilization of pre-existing polar domains and/or transformation of marginal *m*-phase grains under reduced defect kinetics. At room temperature, oxygen vacancies (V_O_) and other mobile point defects can screen the polarization and promote relaxation toward the centrosymmetric *m*-phase during cycling. Cooling to 10 K suppresses defect migration and detrapping, strengthening the local impact of applied electric field during wake-up to align dipoles and lower the free energy of polar variants relative to the *m*-phase without being counteracted by rapid defect reconfiguration. The ultrathin geometry further amplifies interfacial field and elastic boundary conditions that are known to favor *Pca*2_1_ in hafnia-based ferroelectrics.^30–32,40^

Independent chemical evidence from XPS supports this interpretation (Figure 2a,2b). High-resolution O 1s spectra, consistently deconvoluted into lattice oxygen (∼530.0 eV), vacancy/suboxide–related oxygen (∼531–532 eV), and adsorbates at higher binding energies, reveal a clear temperature-dependent redistribution of spectral weight. As the temperature decreases from 300 to 10 K, the lattice-oxygen component progressively intensifies while the defect-related contribution diminishes. This trend reflects strengthened metal–oxygen coordination and suppressed oxygen-vacancy activity under cryogenic conditions. Quantitative analysis of the fitted peak areas is summarized in Figure 2c. The lattice oxygen fraction increases from 57.4% at 300 K to 68.9% at 10 K, while the defect-related oxygen fraction decreases from 27.5% to 14.0%. These values were obtained by integrating the spectral intensity within defined binding-energy windows (529.5–530.5 eV for lattice oxygen, 531–532 eV for defect oxygen) and normalizing to the total O 1s envelope (528–533 eV). The observed redistribution supports the hypothesis that cryogenic wake-up stabilizes the polar orthorhombic phase by reducing suboxide formation and enhancing lattice ordering.

**Figure 2:**
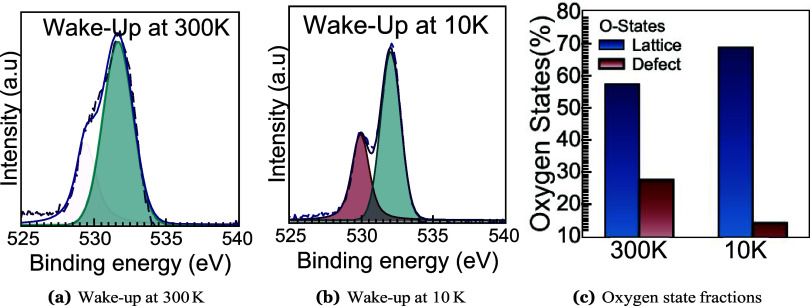
Temperature- and cycling-dependent O 1s chemistry in ultrathin HZO. (a–b) Post–wake-up high-resolution O 1s XPS spectra at 300 and 10 K, each fit with identical components: lattice oxygen (M–O), defect/oxygen-vacancy–related oxygen (*V*_O_/suboxide), and adsorbates. (c) Quantified oxygen state fractions extracted from XPS peak fitting, showing increased lattice oxygen and reduced defect oxygen at 10 K, consistent with enhanced metal–oxygen coordination and cryogenic stabilization of the ferroelectric phase.

Taken together, the structural (4D-STEM) and chemical (XPS) data establish a coherent understanding of the physical mechanism for the device-level improvements measured at 10 K. The larger *o*-phase fraction provides a bigger reservoir of switchable polarization, directly translating to a wider and more stable MW in the FeFET transfer characteristics. Suppressed defect mobility at cryogenic temperature (10 K) reduces both internal screening and back-switching, improving the *V*_th_ distributions and slowing time-dependent drift.

In summary, nanoscale phase mapping reveals that electrical wake-up at cryogenic temperature (10 K) increases the polar orthorhombic fraction from 21.4% (300 K) to 34.9% (10 K) in ∼5 nm HZO, while XPS indicates strengthening of metal–oxygen bonding and a reduction in defect-related oxygen signatures. These complementary observations provide direct, materials-level insights on the physical mechanism behind improved ferroelectric *Pca*2_1_ polymorph and suppresses defect activity at 10 K, thereby explaining the enhanced switching robustness, larger nonvolatile window, and improved state stability observed in our low-temperature FeFETs.

### Polarization Response of Ferroelectric Gate-Stack at 300 K and 10 K

To directly probe the switching dynamics of the ferroelectric gate stack, we performed positive-up-negativ-down (PUND)-based electrical measurements at 300 and 10 K (Figure 3). The gate waveform and current response (Figure 3a) enable extraction of the net switching current by subtracting nonswitching contributions: (*I*_P_ – *I*_U_) for positive and (*I*_N_ – *I*_D_) for negative polarization reversal. The corresponding switching polarization *P*_SW_ is obtained via time-domain integration normalized to the device area. Temperature-dependent switching current density *J*_SW_ (Figure 3b) reveals a significant reduction in peak values at 10 K, consistent with suppressed domain-wall mobility, slower polarization kinetics (*J*_SW_ ∝ d*P*/d*t*), and the freezing out of defect-assisted conduction pathways. The extracted polarization loops (Figure 3c) show diminished remanence and narrowed hysteresis at 10 K, indicating incomplete domain reversal and stronger pinning. Additionally, the gate capacitance *C*_G_ versus *V*_G_ (Figure 3d) exhibits reduced hysteresis and enhanced imprint at low temperature, further supporting the need for elevated operating fields to achieve full switching under cryogenic conditions.

**Figure 3:**
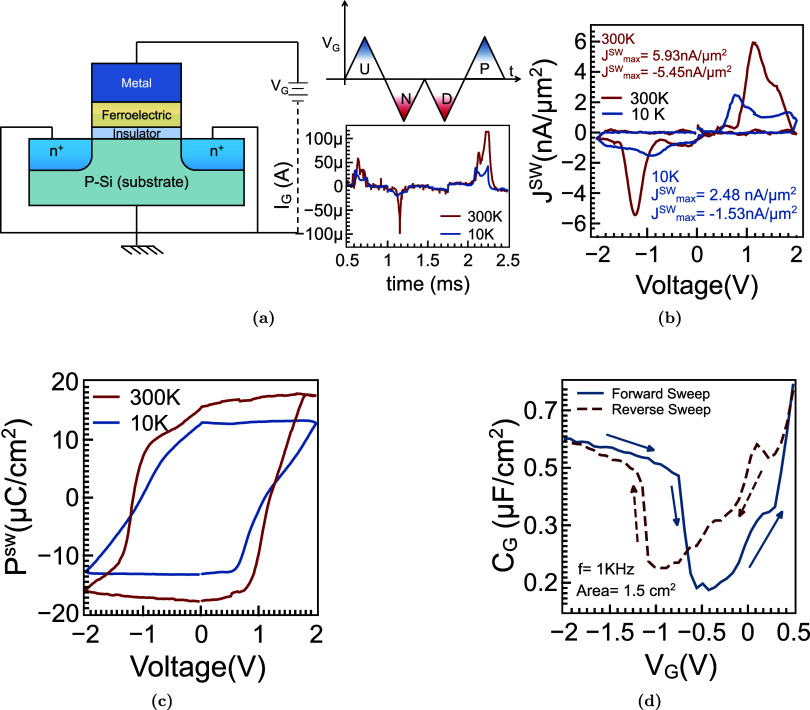
PUND-based electrical characterization of ferroelectric gate stack in FeFET. Gate voltage waveform applied to the ferroelectric gate stack with all terminals grounded, illustrating the extraction of switching currents. The total switching current is obtained as the time-series sum of (*I*_P_ – *I*_U_) for positive-direction switching and (*I*_N_ – *I*_D_) for negative-direction switching. The corresponding switching polarization is calculated as $P_{N}=\frac{1}{A}\int_{t_{1}}^{t_{2}}I_{\mathrm{SW}}\left(t\right)dt$ , where *A* is the device area (b) temperature-dependent switching current density (*J*_SW_) versus gate voltage at 300 and 10 K. The reduced peak currents at cryogenic temperatures arise from suppressed domain-wall mobility, slower polarization kinetics (*I* ∝ d*P*/d*t*), and the freezing out of defect-assisted conduction pathways that contribute at room temperature. (c) Extracted switching polarization (*P*_SW_) from the negative loop, showing diminished remanence and narrowing of the hysteresis at 10 K. This reduction reflects incomplete domain reversal due to stronger pinning, alongside an increased coercive field requirement at low temperature, meaning higher voltages are needed to fully switch polarization. (d) Gate capacitance (*C*_G_) versus gate voltage under forward and reverse sweeps at 1 kHz, revealing dielectric nonlinearity and ferroelectric imprint. The narrowing of the capacitance hysteresis at 10 K supports the need for higher operating field under cryogenic conditions.

### PG/ER Operations of FeFETs at 300 K and 10 K

We first assess the switching and memory behavior of the fabricated FeFETs at 300 K and 10 K (Figure 4). Transfer characteristics *I*_D_–*V*_G_ measured by slowly varying *V*_G_ from −0.5 to 2 V at *V*_DS_ = 0.1 V after PG/ER operation, show a monotonic threshold shift as the program-pulse amplitude is increased from *V*_P_ = 2 to 6 V (rectangular pulses, *t*_pw_ = 500 ns). The extracted *V*_th_ (constant-current criterion of 10 μA) moves from ∼0.1 V (LVT, 6 V) to ∼0.9 V (HVT, −6 V), yielding a MW of Δ*V*_th_ = *V*_th,HVT_ – *V*_th,LVT_ approaching ∼0.8 V (Figure 4a). The smooth evolution of *V*_th_ with *V*_P_ indicates reproducible polarization reversal in the ∼5 nm HZO film under room-temperature conditions. Programming-speed sweeps at fixed *V*_P_ = 5 V identify a minimum effective pulse duration of *t*_pw,min_ ≈ 900 ns for stable nonvolatile switching (Figure 2b). For *t*_pw_ < 900 ns the resulting Δ*V*_th_ diminishes rapidly, consistent with nucleation-limited dynamics in ultrathin hafnia wherein incomplete domain growth and back-switching dominate when the field is not applied long enough to overcome interfacial depolarization and defect pinning.

**Figure 4:**
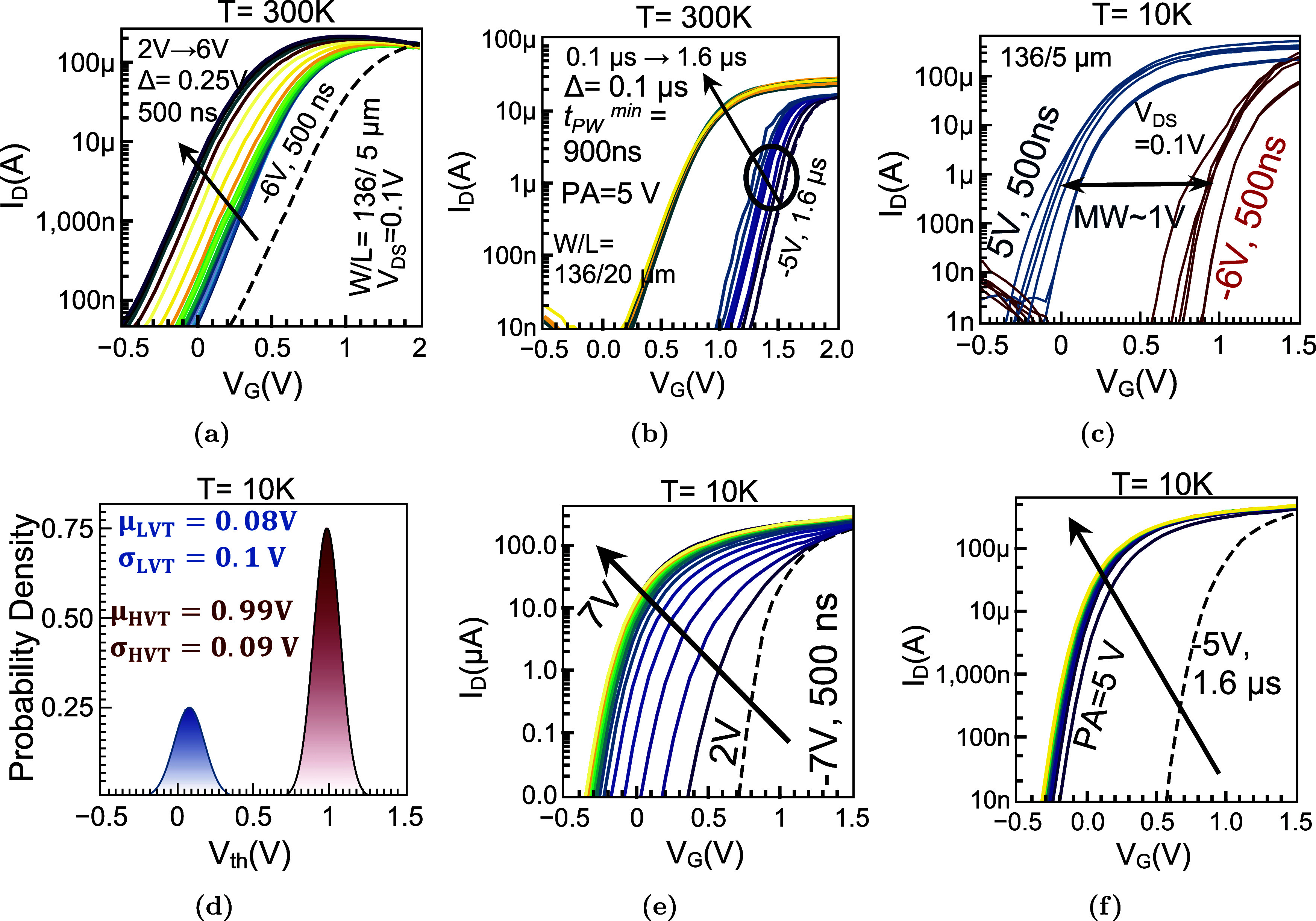
PG/ER Operation of FeFETs with ∼5 nm HZO at 300 K and 10 K. (a) Transfer characteristics *I*_D_(*V*_G_) following programming with gate-voltage amplitude *V*_P_ = 2–6 V in steps of Δ*V*_P_ = 0.25 V (rectangular pulses, *t*_pw_ = 500 ns), showing a monotonic threshold-voltage modulation Δ*V*_th_ consistent with ferroelectric polarization switching. (b) Programming-speed study at *V*_P_ = 5 V: the minimum pulse width producing a stable, nonvolatile shift is *t*_pw,min_ ≈ 900 ns, extracted from sweeps with *t*_pw_ = 0.1–1.6 μs in steps of Δ*t*_pw_ = 0.1 μs. (c) *I*_D_–*V*_G_ characteristics after a program/erase (PG–ER) cycle with *t*_pw_ = 500 ns rectangular pulses. Curves illustrate nonvolatile, polarization-controlled modulation of channel conductance; the memory window (MW), defined as Δ*V*_th_ = *V*_th,HVT_ – *V*_th,LVT_, exceeds 1 V under low read bias. Threshold voltages are extracted by a constant-current criterion (Methods section). (d) Distributions of *V*_th_ across *n* = 8 devices for both states at 10 K show a narrow spread and a clear separation, indicating low device-to-device variability of the polarization-set surface potential. (e) Programming-amplitude sweep: increasing the gate-pulse amplitude *V*_P_ enlarges Δ*V*_th_ until a saturation regime beyond ∼5 V, where additional field does not produce a proportionate gain, consistent with near-complete fer-roelectric switching within the 5 nm HZO. (f) Programming-speed sweep: MW grows with pulse width and saturates for *t*_pw_ ≳ 900 ns, indicating that switching kinetics at 10 K are limited by field-assisted processes and that longer pulses yield diminishing returns. Incremental sequences in *V*_P_ and *t*_pw_ demonstrate reproducible, nonvolatile state placement without drift across repeated cycles, while also showing that moderately elevated *V*_P_ (and subμs pulses near the saturation regime) provide the best trade-off between window size and stress under cryogenic operation.

PG/ER experiments performed at 10 K establish that the ultrathin HZO FeFETs switch robustly and reproducibly under modest drive voltage. A single PG–ER operation with pulses of 5 V/–6 V and t_pw_ = 500 ns produces well-separated LVT/HVT states, with a memory window MW = Δ*V*_th_ > 1 V (Figure 4c).

The statistical quality of the FeFETs under cryogenic conditions is quantified in Figure 4d shows probability density functions (PDFs) of *V*_th_, compiled across devices, reveal narrow unimodal distributions for both LVT/HVT. The device-to-device (D2D) standard deviations are small for each state, yielding no overlap between the PDFs. Such tight distribution of *V*_th_ at 10 K is consistent with a reduction (not complete elimination) in thermally assisted charge trapping in the ferroelectric/oxide stack: lower temperature suppresses emission from shallow traps and slows the redistribution of interfacial charge (Figure S3), thereby stabilizing the polarization-set surface potential.

Comprehensive amplitude and pulse width sweeps define the design window for cryogenic operation. In Figure 4e, the MW increases monotonically as the programming voltage *V*_P_ is raised from 2 V and saturates for |*V*_P_| ≈ 5 V at a fixed pulse width of *t*_pw_ = 500 ns, beyond which further gains are negligible. Similarly, Figure 4f show the pulse-width dependence at fixed |*V*_P_| = 5 V, where the MW increases with *t*_pw_ = 500 from 1.6 μs and plateaus for *t*_pw_ ≥ 0.9 μs. The joint saturation with amplitude and time is indicative of nucleation-limited polarization reversal in ultrathin hafnia films, where a finite density of activation sites and domain-wall pinning centers sets the rate and extent of switching at 10 K. Once a path of switchable domains is established, additional field or pulse time primarily drives subcritical wall motion that contributes little to the macroscopic *V*_th_ shift. Taken together, the 10 K characterization yields clear biasing rules for reliable and energy-efficient operation, aligned with the measured polarization saturation and switching transients, and they reconcile the benefits of cryogenic operation (reduced trap activity, better electrostatics) with the realities of scaled EOT and short-channel effects.

### Reliability of FeFETs at 300 K and 10 K

A comprehensive reliability screen at 10 K exposes endurance and retention boundaries that are set by trap-driven electrostatics rather than by intrinsic ferroelectric (FE) fatigue. Figure 5a shows the waveform for endurance and retention characteristics. Endurance measurements with symmetric stress of +5/–5 V, *t*_pw_ = 500 ns (read at *V*_DS_ = 0.1 V) show well-separated HVT/LVT states with MW *>* 0.5 V for ∼10^3^ cycles (Figure 4b). Beyond 10^4^ cycles, the window contracts and the HVT state drifts toward the LVT state, behavior attributable to charge injection into HZO/SiO_2_ traps and partial polarization screening. The endurance trend at 300 K therefore reflects a balance between robust ferroelectric switching and progressive trap-assisted degradation under repeated high-field pulsing. Room-temperature retention further exposes the role of defect kinetics: both *V*_th,LVT_ and *V*_th,HVT_ relax toward intermediate values on logarithmic time scales, with stable separation not sustained beyond ∼10^5^ s (Figure 5c). We attribute this to thermally activated trapping of holes and internal field screening, which promote slow back-switching. At 10 K, FeFETs sustain ∼10^7^ PG–ER cycles while maintaining clearly resolvable low- and high-threshold states (Figure 5d,e). The apparent endurance ceiling arises from measurement time and storage constraints, not from device degradation. Throughout cycling, both the low- and high-threshold voltages exhibit a monotonic decrease with increasing cycle count. This parallel negative drift is primarily indicative of net positive trapped charge accumulation (e.g., holes injected during negative pulses or donor-like defects) at the ferroelectric/interfacial layers. Contributions from polarization fatigue or built-in field relaxation may account for the narrowing of the MW. At cryogenic temperature (10 K), suppressed thermal detrapping prolongs charge retention, exacerbating the screening effect and symmetric degradation. The gradual *V*_th_ evolution observed during endurance and retention measurements at 10 K does not indicate ferroelectric degradation or thickness-driven fatigue. Instead, it arises from cumulative interfacial charge trapping during repeated read operations. Because detrapping kinetics are strongly suppressed at cryogenic temperatures (10 K), injected charge accumulates and progressively shifts *V*_th_ over time. These observations are consistent with the freezing of thermally activated depolarization and trap emission at low *T*, which slows the detrapping kinetics that typically erode the MW at 300 K.

**Figure 5:**
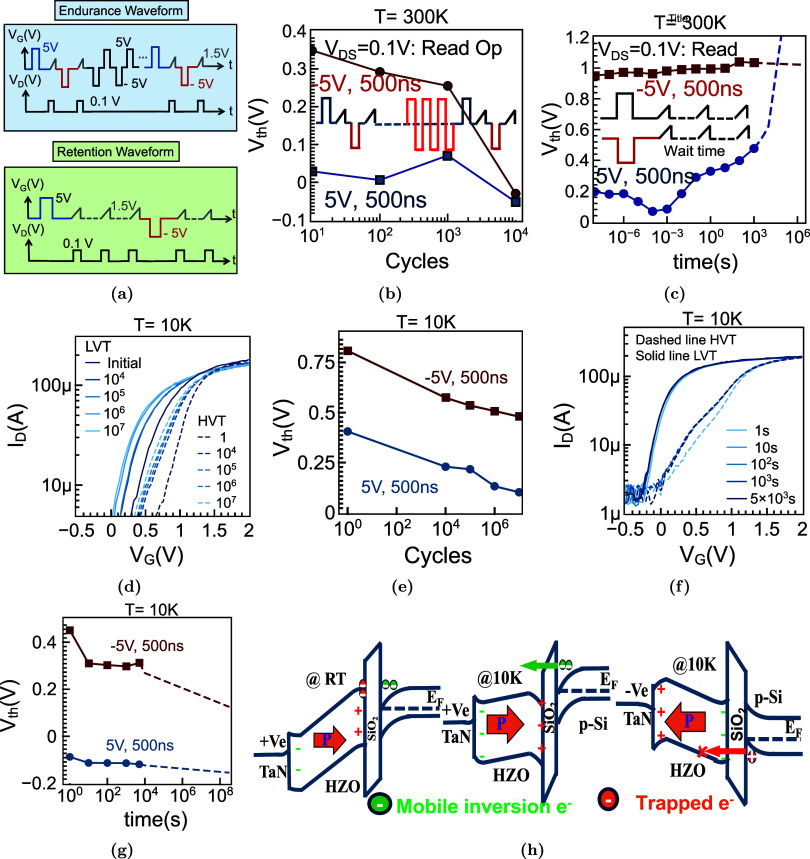
Reliability of FeFETs at 300 and 10 K. (a) Schematic of the test setup. (b) Endurance under symmetric stress of +5/–5 V, 500 ns pulses for 10^4^ cycles; the MW narrows at RT, consistent with charge trapping and partial back-switching in thin films. (c) RT retention of programmed HVT/LVT states for sub-5 nm HZO FeFETs, showing drift in *V*_th_ over time. (d, e) Transfer curves showing LVT (solid) and HVT (dashed) states during cyclic programming. The MW narrows gradually but remains clearly resolvable up to 10^7^ cycles. (f, g) *Retention characteristics*: LVT/HVT states show minimal drift over logarithmic time after PG/ER operations. Extrapolation supports >10-year stability at 10 K, consistent with suppressed thermally activated depolarization and trap emission. (h) *Band-diagram summary:* Under gate bias, polarization in HZO points toward/outward the SiO_2_/p-Si interface, inducing mobile inversion electrons/accumulation holes in the p-Si channel. At room temperature, thermionic generation and trap emission can destabilize the polarization-set surface potential. In contrast, at 10 K, carrier freeze-out and suppressed trap kinetics stabilize the inversion layer, enabling robust readout.

### Atomistic Modeling of a-SiO_2_/HZO Interface

The optimized structure, shown in Figure 6(a,b), was used as the input for molecular dynamics (MD) simulations conducted at 300 K for 0.5 ps. During this equilibration phase, both potential energy and temperature were stabilized using the MACE potential.^50,51^ The equilibrated atomic configuration and its corresponding band diagram are presented in Figure 6(c),(d), respectively. Subsequently, the system was cooled to 10 K to emulate cryogenic conditions and simulated for an additional 10 ps. The final structure and its band diagram under cryogenic operation are shown in Figure 6(e),(f).

**Figure 6:**
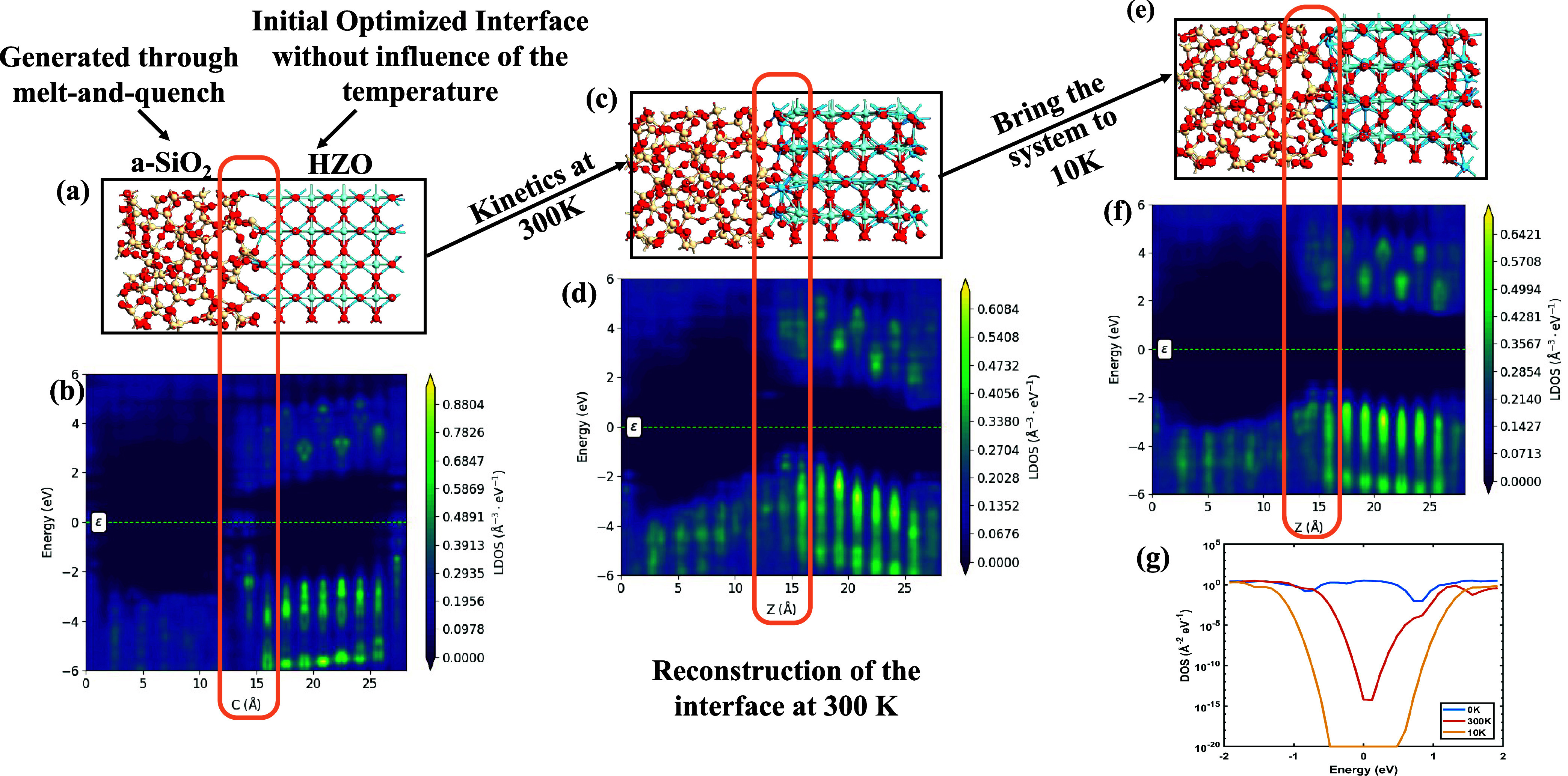
Atomistic modeling of the a-SiO_2_/HZO interface. (a) Optimized structure of orthorhombic HZO with amorphous SiO_2_. (b) Local-density-of-states (LDOS) analysis and band diagram at 0 K obtained from DFT. (c) SiO_2_/HZO structure after MD equilibration at 300 K. (d) LDOS analysis and band diagram at 0 K obtained from DFT for the 300 K structure. (e) Final structure after cooling to 10 K via MD simulation. (f) Corresponding LDOS and band diagram at 10 K.

Figure 6(g) compares the interface density of states (DOS) for the initial structure, after equilibration at 300 K, and after cooling to 10 K. A clear reduction in interface trap states is observed at cryogenic temperatures (10 K). This reduction is significant because interface traps play a crucial role in degrading the stability of ferroelectric polarization in HZO. At higher trap densities, the polarization is partially screened, which weakens the control of HZO over the HVT and LVT states. By lowering the temperature to 10 K, the suppression of these traps leads to stronger polarization coupling and improved separation between HVT and LVT. Moreover, interface traps are a well-known source of read disturbance in ferroelectric devices, as they facilitate charge trapping/detrapping during read operations. Their reduction at cryogenic temperatures (10 K) therefore results in diminished read disturbance, which is directly reflected in the improved transfer characteristics.

Supporting measurements on Al_2_O_3_ reference stacks (see Supporting Figure S4) confirm that the observed cryogenic stabilization is also possible for HZO-FeFETs with Al_2_O_3_ interfacial layers. However, a detailed study on the physics is necessary to further understand the role of the interfacial layer’s chemistry on the reliability of the FeFETs.

### Cryogenic FeFET Synapses for Neuromorphic Inference

We evaluated the suitability of FeFET synapses for neuromorphic inference using both static and event-driven benchmarks. A two-layer spiking neural network (SNN) was first trained on a five-class MNIST task using a device-aware FeFET synapse model directly parametrized from measured electrical characteristics (Figure 7a). The network comprises 784 leaky-integrate-and-fire (LIF) input neurons, lateral inhibition in the output layer, and a winner-takes-all decision rule. Synaptic efficacy is encoded in discrete threshold-voltage states programmed via ±5 V pulses (*t*_pw_ ≈ 900 ns) and read at low drain bias to minimize read-disturb. Training is performed using spike-timing-dependent plasticity (STDP) with clipped multilevel weights, while device-to-device variability, cycle-to-cycle variation, and endurance constraints are explicitly incorporated.^52^

**Figure 7:**
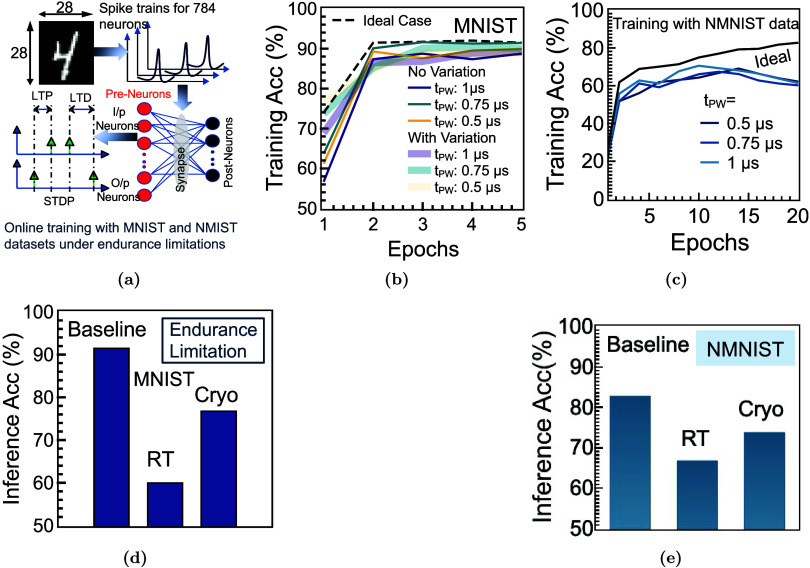
Cryogenic FeFET synapses for neuromorphic inference. (a) Schematic of the device–algorithm mapping used to evaluate FeFETs as nonvolatile synapses in a spiking neural network (SNN) on the MNIST and NMNIST digit-classification task. The synaptic weight is encoded by the FeFET threshold state set via program/erase (PG–ER) pulses, while readout is performed using a low-bias *I*_D_–*V*_G_ transfer. Network-level simulations employ a device-aware model parametrized by measured cryogenic (10 K) switching curves and read characteristics (Methods section), capturing the finite memory window, state drift, and device-to-device variability. (b, c) Training accuracy for MNIST and NMNIST data sets, considering endurance constrained, as a function of programming pulse width *t*_pw_ and imposed device variation, showing >92% accuracy over a broad operating range. (d, e) Superior inference accuracy is observed under cryogenic operation due to lower device variation.

Under endurance-limited conditions, the MNIST network converges reliably and maintains stable accuracy across training epochs (Figure 7b). To assess performance on event-driven data, we extended the evaluation to the N-MNIST data set using a three-layer fully connected SNN implemented in snnTorch (Figure 7d). Flattened 34 × 34 × 2 event streams (2312 input neurons) project to a hidden layer of 1000 LIF neurons and a 10-neuron output layer. Synaptic weights are mapped to analog FeFET threshold states, incorporating quantization and endurance-induced degradation calibrated from experimental measurements. The impact of programming pulse width and amplitude on classification accuracy is systematically examined.

While the ideal software baseline converges to ∼83.1% accuracy, endurance degradation at room temperature reduces the best last-epoch accuracy to 66.8%, underscoring the sensitivity of event-based inference to synaptic reliability. In contrast, cryogenic operation at 10 K consistently enhances inference robustness across both benchmarks: MNIST accuracy exceeds 92% under endurance constraints (Figure 7c), and N-MNIST accuracy recovers to 73.8% (Figure 7e), representing a 7% improvement over room temperature.

This cryogenic advantage arises from three key mechanisms: (i) a larger and more stable memory window, improving separability of discretized weights; (ii) superior endurance and retention; and (iii) tighter *V*_th_ distributions, which suppress accumulated quantization noise. These results validate FeFET synapses as robust primitives for low-temperature, energy-efficient neuromorphic inference and highlight the importance of codesigning training protocols with experimentally measured cryogenic switching and reliability envelopes.

## Discussion

This work presents a comprehensive experimental investigation of aggressively thickness-scaled FeFETs with sub-2 nm EOT gate stacks, focusing on their electrical behavior, reliability limits, and defect dynamics under cryogenic operation. Rather than positioning the devices as a fully optimized ferroelectric memory benchmark, our results provide a physics-based materials- and device-level study that elucidates how ultrathin HZO films, interfacial dielectric choice, and low-temperature defect kinetics jointly govern FeFET performance. A sub-2 nm equivalent-oxide-thickness (EOT) stack by integrating an ultrathin (∼5 nm) Hf_0.5_Zr_0.5_O_2_ (HZO) layer within a high-*k*/metal-gate architecture, and evaluate device behavior down to 10 K. At this temperature, the transistors sustain a MW > 1 V, endurance >10^7^ program/erase (PG–ER) cycles under low-disturb read bias, and projected retention >10 years. These figures-of-merit are achieved with modest programming conditions—rectangular pulses of ±5 V and *t*_pw_ ≈ 500–900 ns—so the total write energy remains in the subfemtojoule range per bit.

The central enabling mechanism is a materials–bias codesign that stabilizes the ferroelectric orthorhombic phase (*Pca*2_1_) at cryogenic temperature (10 K) while maintaining strong electrostatics in the aggressively scaled gate stack. Correlative structural analysis confirms this picture. Automated crystal orientation mapping (ACOM) on 4D-STEM data sets reveals an increase of the orthorhombic phase fraction after electrical “wake-up” at 10 K, relative to room temperature, and high-resolution X-ray photoelectron spectroscopy (XPS) shows a consistent strengthening of lattice-oxygen signatures. Together, these data point to a cryo-induced reduction in defect participation and a more stable polarization landscape in ultrathin HZO. On the device side, these structural changes manifest as tighter *V*_th_ statistics across devices and cycles, steeper cumulative distribution functions, and an MW that is resilient to modest programming variations.

Electrical characterization establishes practical operating recipes. First, switching saturates at ±5 V: increasing |*V*_P_| to ±7 V or extending *t*_pw_ beyond ∼900 ns yields only marginal gains in Δ*V*_th_. A notable outcome of deep-cryogenic operation is the decoupling of several room-temperature trade-offs. Reduced thermal activation at 10 K suppresses stochastic charge exchange with shallow traps and slows emission from deeper levels, which in turn improves device-to-device *V*_th_ spreads. At the same time, the sub-2 nm EOT stack retains strong gate control, so depolarization fields do not collapse the ferroelectric state despite the ultrathin interfacial layer. The combined effect is a window of operating conditions where MW, variability, and endurance are simultaneously favorable—a prerequisite for large-array deployment near the quantum stack.

Beyond immediate device metrics, these results have system-level implications. The FET-native read path enables nanosecond-class sensing without the current integration or destructive read inherent to many alternative memories, and the sub-fJ write energy minimizes heat deposition on cold stages. In cryogenic control electronics—where wiring count, thermal budget, and calibration stability dominate—the ability to store state locally with negligible standby power is particularly attractive. Moreover, the device characteristics demonstrated here map naturally to in-memory compute primitives: small-signal charge-sharing or current-mode multiply–accumulate operations can be executed with low *V*_DS_ to avoid disturb, leveraging the nonvolatility of the ferroelectric polarization to hold weights.

The comparison table (Table 1) provides a comparative overview of recent cryogenic studies on HfO_2_-based ferroelectric devices, highlighting the diversity of experimental platforms and reported performance metrics. Our work on bulk Si FeFETs with a scaled HZO layer (∼5 nm, sub-2 nm EOT) demonstrates a memory window exceeding 1 V and endurance beyond 10^7^ cycles over the temperature range from 300 K down to 10 K. These electrical characteristics are further supported by structural and low-frequency noise analyses, which elucidate the underlying switching behavior and trapping dynamics at cryogenic temperature (10 K).

**Table 1:** Benchmarking of Representative Cryogenic HfO_2_-Based FeFETs Compared with This Work^a^

metric	this work	VLSI 2023 ^23^	TED 2024 ^53^	APL 2020 ^42^	IMW 2024 ^34^
Device/Platform	Bulk-Si FeFET	W–In_2_O_3_ FeFET (BEOL)	FDSOI Si-FeFET	FDSOI Si-FeFET	FeFET
CMOS compatibility	HKMG on 200 mm/Si platform	BEOL demonstrator	FDSOI platform	FDSOI platform	–
FE material	HZO	HZO	HZO	Si/HfO_2_	Si/HfO_2_
FE thickness	∼5 nm	∼10 nm	9 nm	10 nm	10 nm
EOT/IL	<2 nm EOT	–	–	–	–
Temp range	300 K → 10 K	300 K → 77 K, 5 K	300 K → 5 K	300 K → 6.9 K	2.5 K → 358 K
MW at cryo	>1 V@10 K	1.63× (at 5 K)	Multilevel MW	∼1 V increase	2.3 V@2.5 K
Endurance at cryo	>10^7^@10 K	≥10^10^@5 K	>10^5^@5 K	–	*>*10^4^@2.5 K
Key takeaway	Sub-2 nm EOT bulk Si-FeFET functional at 10 K with >1 V MW and *>*10^7^ endurance	Extreme endurance at 5 K (BEOL FeFET)	Cryo multilevel operation	Cryo MW increase (FDSOI)	Operation down to 2.5 K

a “–” indicates not reported.

In contrast, the Cold-FeFET reported at VLSI 2023 ^23^ employs a W–In_2_O_3_ channel in a BEOL-compatible architecture, achieving effectively unlimited endurance and fast write operation at 77 and 5 K. The TED 2024 study on FDSOI FeFETs^53^ with a 9 nm HZO layer emphasizes multilevel memory operation and stable retention down to 5 K, while earlier work reported in APL 2020 ^42^ on Si/HfO_2_ FDSOI devices observed an ∼1 V increase in memory window upon cooling to 6.9 K, attributed to an increase in the coercive field.

Capacitor-level investigations have also demonstrated exceptional cryogenic reliability. For example, the JXCDC 2021 study^54^ established endurance beyond 3.5 × 10^10^ cycles with negligible fatigue at 4 K, and the Advanced Electronic Materials 2024 report^55^ demonstrated analog ferroelectric operation with up to 20 reproducible states and polarization values reaching 75 μC cm^–2^ at 4 K. Most recently, IMW 2024 ^34^ presented Si/HfO_2_ FeFETs incorporating SiON/SiO_2_ interfacial layers, achieving a 2.3 V memory window at 2.5 K, a low subthreshold swing of ∼40 mV dec^–1^, and stable retention governed by trap dynamics. Taken together, the compact 4*F*2 footprint, sub-fJ energy per operation, competitive latency, high endurance under low-disturb read conditions, and excellent cryogenic stability position Si-based hafnia-based FeFETs as leading candidates for dense, energy-efficient memory and mixed-signal in-memory computing near the quantum stack.

## Conclusion

In this work, we have systematically investigated the cryogenic behavior of ferroelectric field-effect transistors (FeFETs) based on ultrathin HZO layers, combining structural, chemical, electrical, and atomistic analyses with neuromorphic benchmarking. Cross-sectional TEM and 4D-STEM ACOM established the coexistence of monoclinic and orthorhombic polymorphs, with cryogenic wake-up driving a measurable increase in the polar orthorhombic fraction. Complementary XPS confirmed strengthened metal–oxygen coordination and reduced defect-related oxygen signatures at 10 K, highlighting suppressed vacancy activity and enhanced lattice ordering. Together, these materials-level insights explain the improved switching robustness and stability observed in device-level measurements. Electrical characterization using PUND, PG/ER sweeps, and reliability screens revealed that cryogenic operation suppresses defect-assisted conduction, reduces back-switching, and narrows *V*_th_ distributions. Endurance and retention tests demonstrated that trap-driven degradation dominates at room temperature, whereas at 10 K the freezing of defect kinetics prolongs charge retention and stabilizes the nonvolatile window. Atomistic modeling of the SiO_2_/HZO interface further supported these findings, showing a reduction in interface trap states under cryogenic conditions, which mitigates read disturbance and strengthens polarization coupling. Finally, neuromorphic inference experiments using MNIST and N-MNIST benchmarks validated FeFET synapses as robust primitives for low-temperature spiking neural networks. Cryogenic operation consistently improved inference accuracy, sustained endurance-constrained training, and reduced variability, underscoring the synergy between device physics and algorithmic performance.

Overall, this study provides a coherent framework linking cryogenic defect physics, phase stability, and interface trap suppression to enhanced FeFET reliability and neuromorphic functionality. These results not only advance the fundamental understanding of hafnia-based ferroelectrics at low temperature but also establish design rules for cryogenic memory and energy-efficient neuromorphic hardware, where robust polarization control and minimized variability are essential.

## Methods

### Fabrication and Characterization

Solid–solution hafnia–zirconia (SS–HZO) gate dielectrics were grown by thermal atomic layer deposition (ALD) using alternating HfO_2_ and ZrO_2_ cycles on Si/SiO_2_ substrates. Calibrated growth rates were 0.96 Å/cycle (HfO_2_) and 0.92 Å/cycle (ZrO_2_). The supercycle was balanced to achieve a 1:1 Hf/Zr cation composition, yielding a total physical thickness of 5 nm. The gate stack for all FeFETs was TaN/SS–HZO/SiO_2_/p–Si in a gate-first flow. SiO_2_ was selected as the interfacial layer because it provides a chemically stable, nonreactive interface with HZO, minimizes oxygen scavenging, and maintains well-defined band offsets. Unlike alternative high-k ILs such as Al_2_O_3_ or SiON, SiO_2_ does not introduce additional dipoles or mobile ionic species or additional fixed charges, allowing the intrinsic ferroelectric and cryogenic properties of HZO to be isolated. To verify this choice experimentally, we fabricated and evaluated AlO-based FeFETs. Postdeposition rapid thermal annealing (RTA) in Ar was used both for dopant activation and to crystallize the SS–HZO. The process flow of fabrication is shown in Figure 1a. Cross-sectional transmission electron microscopy (TEM) confirmed layer continuity and thicknesses of the TaN/SS–HZO/SiO_2_ stack (Figure 1b). Phase distributions within the 5 nm SS–HZO were mapped by 4D scanning TEM with automated crystal-orientation mapping (ACOM), allowing spatial discrimination of monoclinic and orthorhombic polymorphs. X-ray photoelectron spectroscopy (XPS) was used to assess cation stoichiometry and oxygen coordination/chemical bonding before and after annealing.

Quasi-static and pulsed measurements were performed using a Keysight B1500A analyzer and B1530A WGFMU, respectively. Unless stated otherwise, transfer characteristics *I*_D_–*V*_G_ were recorded in the programmed (HVT) and erased (LVT) states at 300 and 10 K, using a low drain bias to minimize read-disturb. Endurance was evaluated with symmetric program/erase (PG/ER) stress of ±5 V, 500 ns rectangular pulses (B1530A), with periodic verification sweeps. The threshold voltage *V*_th_ was extracted by the constant-current criterion at *I*_D_ = 0.1 μA × (*W*/*L*), where *W* and *L* are the device width and length. The memory window was defined as Δ*V*_th_ = *V*_th,HVT_ – *V*_th,LVT_. Measurements at additional temperatures were also collected, but are not reported here for brevity.

### Atomistic Modeling

The simulations were performed using the orthorhombic phase of HZO and amorphous SiO_2_. The amorphous SiO_2_ structure was generated via the melt-and-quench technique within molecular dynamics (MD) simulations. Following structural construction and optimization, density functional theory (DFT) calculations were initially carried out. The optimized structure and the corresponding band diagram, obtained from the local density of states (LDOS) analysis in the QuantumATK framework,^51^ are shown in Figure 6(a),6(b). The atomistic modeling presented here does not attempt to compute a full transistor-scale band diagram of the TaN/HZO/SiO_2_/p-Si stack due to limited time and resources available in academia. Instead, it provides local electronic-structure information at the HZO/SiO_2_ interface—including the interface density of states, local band-edge alignment, and trap-state behavior—that governs tunneling pathways and polarization screening.

### Neural Network Simulation

We evaluated classification performance using a current-based, spiking neural network (SNN) that consumes measured FeFET device data as the synaptic primitive. The network implements a fully connected, feedforward topology with global lateral inhibition at the output layer and a pair-based spike-timing-dependent plasticity (STDP) learning rule on excitatory synapses. The input layer comprises 784 afferents (flattened 28 × 28 pixels). The output layer size depends on the task: for five-class recognition, we use either 60 neurons (“Nonlinear Synapse”, device-aware FeFET) or 80 neurons (“Ideal Synapse”, linear reference); for ten-class recognition, we use 80 output neurons. Each input neuron connects excitatorily to every output neuron. Fixed lateral inhibitory synapses mutually couple output neurons to enforce a winner-takes-all (WTA) competition during each stimulus window.

All neurons are leaky integrate-and-fire (LIF). The membrane potential *V* evolves as

1 $$\tau_{m}\frac{dV}{dt}=-\left(V-V_{\mathrm{rest}}\right)+R_{m}I_{\mathrm{syn}}\left(t\right)$$

with threshold *V*_th_, reset to *V*_reset_, and absolute refractory τ_ref_. Pixels are normalized to [0, 1] and converted to inhomogeneous Poisson spike trains whose rates are proportional to intensity. For each image, spikes are presented for a fixed simulation window *T*_img_; within that window, lateral inhibition implements WTA so that the most active output neuron dominates the response. *V*_rest_ denotes the resting (leak) potential to which the membrane relaxes, τ_m_ membrane time constant (s), with τ_m_ = *R*_m_ *C*_m_, *R*_m_ Membrane resistance, and *I*_syn_(*t*) Net synaptic input current (A), i.e., excitatory minus inhibitory drive.

Two synapse models are used. (i) *Ideal Synapse* (reference): weights *w* ∈ [*w*_min_, *w*_max_] follow symmetric, state-independent potentiation/depression with linear update steps; no device noise, drift, or endurance limits are applied. (ii) *Nonideal Synapse* (FeFET): each synapse instantiates a device-aware weight state whose evolution is constrained by measured FeFET characteristics at the target temperature (*T*). Device-to-device and cycle-to-cycle variability are injected as perturbations with mean and standard deviations calibrated from the experiment. An endurance budget *N*_cyc_ is enforced per synapse; beyond this, the update probability is reduced, or the step magnitude is attenuated following the measured cycling trend at the corresponding *T* (endurance-constrained setting). All reads are performed at a fixed, low *V*_DS_ consistent with the cryogenic reliability envelope to suppress read-disturb. Excitatory synapses follow a pair-based STDP

2 $$\Delta w=\{\begin{array}{ll} \eta A_{+}e^{-\Delta t/\tau_{+}}S_{+}\left(w\right) & \mathrm{if}\;\Delta t\equiv t_{\mathrm{post}}-t_{\mathrm{pre}}>0\\ -\eta A_{-}e^{\Delta t/\tau_{-}}S_{-}\left(w\right) & \mathrm{if}\;\Delta t<0 \end{array}$$

where η is a learning-rate factor, (*A*_±_, τ_±_) are amplitude/time constants, and *S*_±_(*w*) are state-dependent scaling terms. For Ideal Synapses, *S*_±_(*w*) ≡ 1. For FeFET synapses, *S*_±_(*w*) are set by the device’s measured potentiation/depression nonlinearity (equivalently, we sample Δ*w* directly from Δ*w*_±_(*w*; ·) as noted above). After each update, *w* is clipped to [*w*_min_, *w*_max_]. Lateral inhibitory synapses are fixed and nonplastic.

During training, each image elicits an output spike raster over *T*_img_. A soft/hard WTA mechanism (global inhibition) suppresses nonwinning units to prevent mode collapse and encourages specialization. After training, each output neuron is assigned the label for which it produced the highest cumulative spike count across the training set. Inference runs with learning disabled; the predicted class is the argmax of output spike counts over *T*_img_.

To study temperature, variability, and endurance, we instantiate FeFET synapses with parameter sets drawn from the measured cryogenic (e.g., 10 K) and room-temperature data sets. Programming amplitude *V*_P_ and pulse width *t*_pw_ are chosen within the experimentally validated envelope (e.g., saturation beyond ∼5 V and ∼900 ns at 10 K); endurance-constrained experiments cap the cumulative number of potentiation/depression events per synapse according to the measured cycling budget. All read operations use a low *V*_DS_ consistent with the reliability maps to minimize MW contraction due to lateral-field-assisted injection and DIBL.

Unless otherwise stated, we train for a fixed number of epochs with a constant time step solver; homeostatic mechanisms (activity regularization and mild weight normalization) are applied to avoid dead units and maintain balanced competition. Reported accuracies are averaged over multiple random seeds (data order and initial weights). We compare (a) device-aware FeFET synapses at cryogenic temperature (10 K), (b) the same at room temperature, and (c) the ideal-synapse reference, under identical SNN hyperparameters and presentation time. In endurance-constrained scenarios, cryogenic FeFET synapses maintain separable states with fewer refreshes, yielding >92% accuracy on MNIST within conservative pulse budgets, whereas the room-temperature baseline degrades under the same update cap.

## Supplementary Material



## Notes

X-ray diffraction (XRD) of the ultrathin HZO film (called out in the Structural Analysis section) (Figure S1); phase-composition summary from ACOM/4D-STEM (orthorhombic fraction comparison: RT vs 10 K vs pristine) (Figure S2); temperature-dependent low-frequency noise (SID vs frequency) at 2 K/20 K/300 K (γ slopes) (Figure S3), and Al_2_O_3_ interfacial-layer reference-stack measurements supporting cryogenic stabilization (Figure S4) (PDF)
