# Supplementary material for: Sub‑2 nm Equivalent-Oxide-Thickness Ferroelectric Transistors for Cryogenic Memory and Computing
Source: ACS Nano. 2026 Mar 31;20(14):10905–18. doi: 10.1021/acsnano.5c16255 (PMC13085857; doi:10.1021/acsnano.5c16255)
Supplement: Supplementary file 1 [file nn5c16255_si_001.pdf]

Sub-2 nm equivalent-oxide thickness ferroelectric transistors for cryogenic memory and computing

Apu Das,[†] Asim Senapati,[†] Gautham Kumar,[†] Zhao-Feng Lou,[‡] Jonas Müller,[¶] Jaskirat Singh Maskeen,[§] Yii-Tay Chang,[‡] Mohit Tewari,[‖] Ankit Agarwal,[⊥] Agniva Paul,[†] Yannick Raffel,[#] Siddheswar Maikap,[@] Kuo-Hsing Kao,[⊥] Tarun Agarwal,[‖] Sandip Lashkare,[‖] Darsen Lu,[*,⊥] Guilhem Larrieu,[*,¶] Min-Hung Lee,[*,‡] and Sourav De[*,†]

†*College of Semiconductor Research (CoSR), National Tsing Hua University (NTHU), Hsinchu 300044, Taiwan*

‡*Graduate Institute of Electronics Engineering (GIEE), National Taiwan University (NTU), Taipei 106319, Taiwan*

¶*Laboratory for Analysis and Architecture of Systems (LAAS-CNRS), Université de Toulouse, 31031 Toulouse, France*

§*Department of Computer Science and Engineering, Indian Institute of Technology Gandhinagar, Palaj, Gujarat 382055, India*

‖*Department of Electrical Engineering, Indian Institute of Technology Gandhinagar, Palaj, Gujarat 382055, India*

⊥*Department of Electrical Engineering, National Cheng Kung University (NCKU), Tainan 70101, Taiwan*

#*Fraunhofer Institute for Photonic Microsystems IPMS – Center Nanoelectronic Technologies, 01109 Dresden, Germany*

@*Department of Electronic Engineering, Chang Gung University (CGU), Taoyuan, Taiwan*

E-mail: darsenlu@mail.ncku.edu.tw; glarrieu@laas.fr; minhunglee@ntu.edu.tw; sourav.de@mx.nthu.edu.tw

Supplementary Information

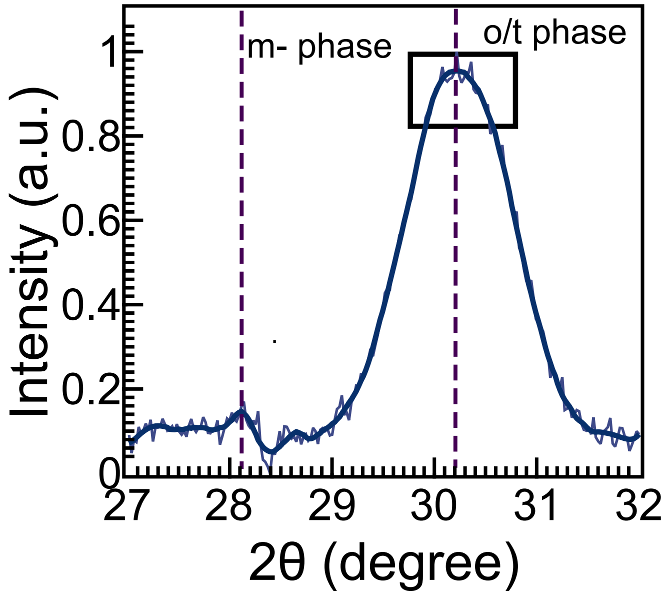

Figure S1: GI-XRD pattern. GI-XRD pattern distinctly resolves characteristic diffraction peaks corresponding to the m-phase and o/t-phase crystal structures.

Phase Identification via Grazing-Incidence X-Ray Diffraction Figure S1 presents the grazing-incidence X-ray diffraction (GI-XRD) pattern of the ultrathin HZO film, measured in the angular range $2\theta = 27°–32°$. The spectrum reveals distinct diffraction features corresponding to the monoclinic (m-phase) and orthorhombic/tetragonal (o/t-phase) crystal structures. A pronounced peak near $2\theta \approx 30°$ is attributed to the o/t-phase, consistent with the (111) reflection of the polar orthorhombic phase and/or the nonpolar tetragonal phase. In contrast, the shoulder near $2\theta \approx 28°$ corresponds to the m-phase, typically associated with non-ferroelectric behavior.

The relative prominence of the o/t-phase peak suggests partial stabilization of the ferroelectric orthorhombic phase, likely induced by interface strain, wake-up cycling, or dopant-mediated phase competition. The use of grazing incidence enhances surface sensitivity, allowing phase identification in ultrathin films where conventional XRD lacks sufficient signal-to-noise ratio. These results confirm the coexistence of multiple HfO_2 polymorphs and support the presence of ferroelectric ordering in the active layer.

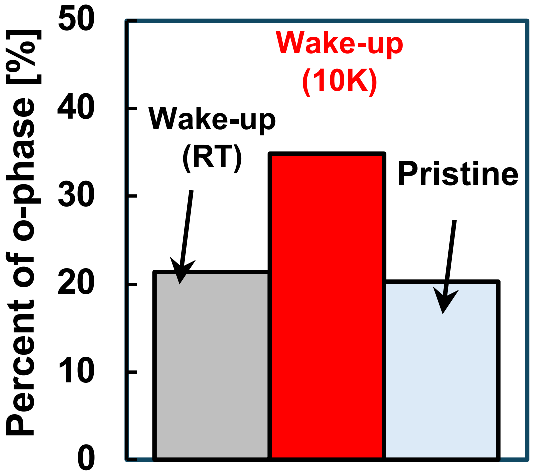

Figure S2: Phase-composition summary extracted from ACOM mapping. Phase-composition quantification over the mapped area underscores enrichment of the ferroelectric *o*-polymorph ($Pca2_1$) after wake-up at 10 K relative to RT and pristine baselines. The enhanced *o*-phase fraction provides a structural basis for the improved cryogenic device characteristics (larger, more stable memory window and tighter V_{th} statistics).

Phase-composition summary from ACOM mapping Figure S2 summarizes the orthorhombic (*o*)-phase fraction extracted from the automated crystal-orientation mapping (ACOM) analysis of the ultrathin (~5 nm) HZO film. The *o*-phase is the ferroelectric polymorph ($Pca2_1$) responsible for switchable polarization in HfO_2-based ferroelectrics. Three representative conditions are compared: the pristine state (as-processed, before electrical conditioning), wake-up at room temperature (RT), and wake-up performed under cryogenic operation at 10 K.

A clear enhancement of the ferroelectric *o*-phase fraction is observed after cryogenic wake-up, rising to ~35% compared with ~20–22% for the pristine and RT wake-up conditions. This trend indicates that electrical conditioning at 10 K more effectively stabilizes the polar orthorhombic phase than wake-up at RT, consistent with a redistribution of phase stability under combined low-temperature operation and electric-field cycling. Importantly, the increase in *o*-phase content provides a structural basis for the cryogenic electrical characteristics reported in the main text, including a larger and more stable memory window and reduced statistical spread in V_{th}. In this interpretation, enrichment of the polar phase improves the effective remanent polarization. It reduces the susceptibility of the switching landscape to local non-ferroelectric regions, thereby supporting more reproducible program/erase behavior at 10 K.

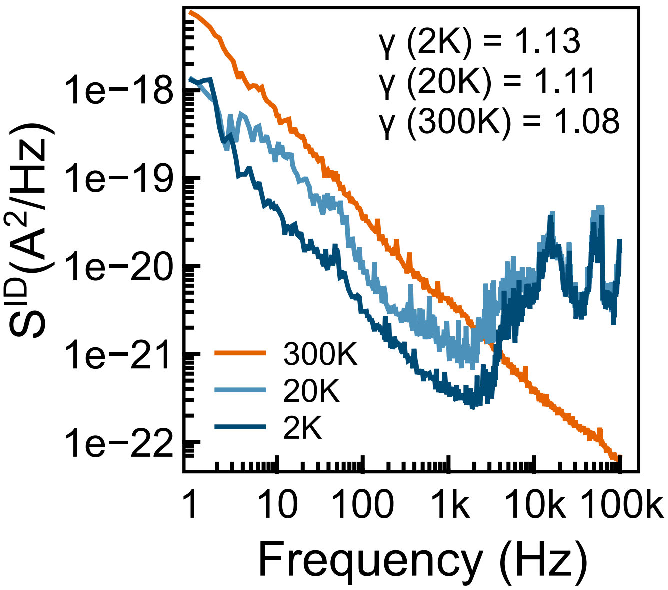

Figure S3: Temperature-dependent low-frequency noise behavior in HSO(10nm) FeFETs. Drain current noise spectral density (S_{ID}) versus frequency measured at 2 K, 20 K, and 300 K. The slope parameter γ extracted from power-law fits increases with decreasing temperature ($\gamma = 1.08$ at 300 K, $\gamma = 1.11$ at 20 K, and $\gamma = 1.13$ at 2 K), indicating a progressively broader distribution of trap time constants. At cryogenic temperatures, suppressed thermal emission and prolonged trap occupancy enhance low-frequency fluctuations, consistent with interface-trap dominated noise. At room temperature, trap dynamics are faster and more uniform, yielding a flatter spectrum closer to ideal $1/f$ behavior.

Temperature-Dependent Low-Frequency Noise Analysis in FeFETs: Figure S3 presents the drain current noise spectral density $S_{ID}(f)$ as a function of frequency across three temperatures: 300 K, 20 K, and 2 K. The spectra exhibit a power-law dependence of the form $S_{ID}(f) \propto 1/f^{\gamma}$, where the slope parameter γ is extracted from log–log fits. A progressive increase in γ is observed with decreasing temperature: $\gamma = 1.08$ at 300 K, $\gamma = 1.11$ at 20 K, and $\gamma = 1.13$ at 2 K. This trend indicates a broadening of the distribution of trap time constants under cryogenic conditions.

At room temperature, thermally activated emission enables rapid trap occupancy and release, resulting in a relatively uniform ensemble of time constants and a near-ideal $1/f$ spectrum. In contrast, cryogenic operation suppresses thermal emission and prolongs trap occupancy, particularly for deep-level and border traps with large activation energies. This leads to enhanced low-frequency fluctuations and a steeper spectral slope, consistent with interface-trap dominated noise mechanisms. The increase in γ reflects the emergence of slow, non-equilibrium trapping dynamics and a wider spread in activation energies, which are not thermally averaged at low temperature.

These results underscore the sensitivity of FeFETs to trap kinetics across temperature and

provide a quantitative fingerprint of the underlying defect landscape. The extracted γ values serve as a diagnostic metric for interface quality and reliability, and highlight the importance of trap engineering for cryogenic memory and neuromorphic applications.

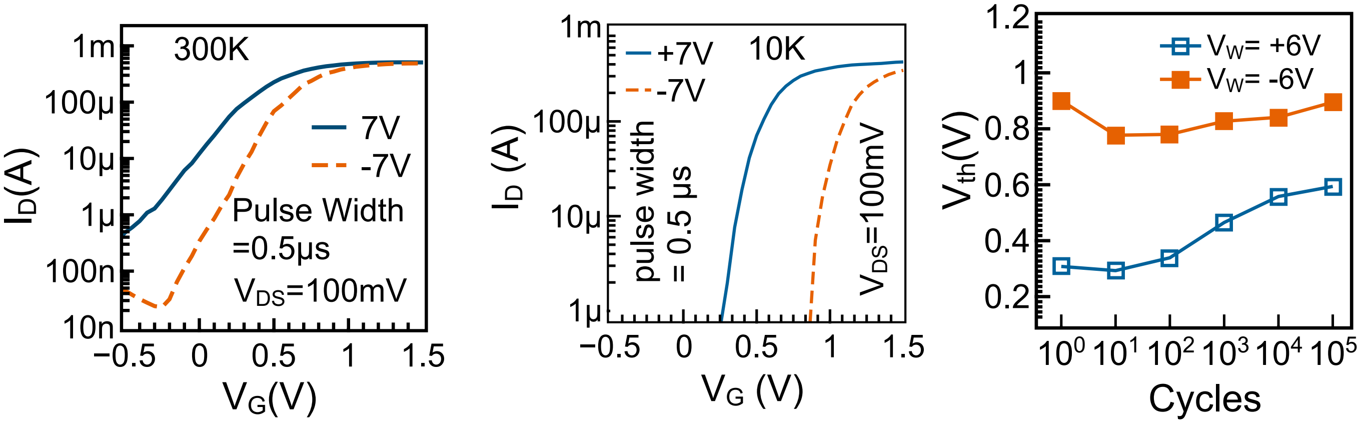

(a) (b) (c)

Figure S4: Cryogenic switching and endurance characteristics of Al_2O_3-interfaced FeFET. (a,b) Transfer characteristics measured at 300 K and 10 K after programming with ±7 V gate pulses (t_{pw} = 0.5 μs, V_{DS} = 100 mV). At 300 K, both LVT (blue, +7 V) and HVT (orange, −7 V) states are well-defined, with clear separation. At 10 K, the memory window remains resolvable, but both states exhibit reduced I_D due to carrier freeze-out and suppressed inversion efficiency. The steeper subthreshold slope at cryo reflects reduced thermal broadening and enhanced electrostatic control. (c) Endurance behavior at 10 K under repeated cycling with ±6 V write pulses. Threshold voltages for LVT (open blue squares) and HVT (filled orange squares) show gradual drift over cycles, with symmetric degradation trends. The parallel shift suggests cumulative trap charging or polarization fatigue, while the preserved window confirms stable switching dynamics of the Al_2O_3 interface stack under cryogenic stress.

FeFET with Al_2O_3 Interface: The Al_2O_3-interfaced FeFETs show different switching behavior at 300 K and 10 K, even though the device structure and interface remain the same. At 300 K (Figure. S4a), transfer curves measured after ±7 V, 0.5 μs programming pulses display a clear memory window between the low-threshold (LVT, +7 V) and high-threshold (HVT, −7 V) states. The subthreshold swing is broadened, which is consistent with thermally activated carrier transport and trap-assisted leakage. At 10 K (Figure. S4b), the programmed states remain well separated, confirming that ferroelectric polarization switching is maintained under cryogenic operation. The subthreshold swing becomes steeper, and the off-state leakage is reduced, reflecting carrier freeze-out and suppressed thermal generation. The memory window remains wide, which indicates that the Al_2O_3 interfacial layer contributes a significant density of traps that continue

to screen the polarization even at low temperature. Endurance data at 10 K (Figure. S4c) show gradual, symmetric threshold-voltage drift for both states during cycling. This parallel shift points to cumulative charge trapping or partial polarization fatigue, while the memory window remains resolvable. In our study, SiO_2-interfaced FeFETs outperform Al_2O_3-interfaced counterparts at 10K; however, further systematic investigation is necessary before drawing definitive conclusions.
